# Excision of the Teeth

**Published:** 1844-09

**Authors:** 


*Excision of the Teeth.*—Dr. Smethurst narrates a case in the London Lancet, where excision of a tooth, a natural one being pivoted on the fang, was followed by violent inflammation and suppuration of the injured part, attended with œdema of the whole head and face, and of the interior of the mouth and tongue, so that the patient was in danger of suffocation. Intense febrile excitement was also present. Bleeding, calomel and acetate of morphia relieved the attack, and, after a time, the pivoted tooth could be removed, when a discharge of pus and blood took place, with considerable relief. Ultimately the health was restored, and the lost tooth made good with a real one fixed with gold wires, which did not occasion any inconvenience whatever.—*Medical Times.*

